# Supplementary material for: A Novel Strategy for Glioblastoma Treatment by Natural Bioactive Molecules Showed a Highly Effective Anti-Cancer Potential
Source: Nutrients. 2024 Jul 23;16(15):2389. doi: 10.3390/nu16152389 (PMC11314145; doi:10.3390/nu16152389)
Supplement: Supplementary file 1 [file nutrients-16-02389-s001.zip › nutrients-3111709-supplementary.pdf]

Table S1

ID	Species	Putative identification	Retention time (min)	Elemental formula	experimental m/z (-)	ESI (-) main adduct	experimental m/z (+)	ESI (+) main adduct	mass error (-). (ppm)	Fragments (-)
1	*A. lusitanicum*	Sinapic acid derivative	4.60	$C_{19}H_{34}O_9$	451.216	$[M+HCOOH-H^+]^-$	429.207	$[M+Na^+]^+$	3.882	De Sorricellis et al., 2024
2	*A. lusitanicum*	Benzylalcohol O-(O-pentosyl-hexoside)	5.08	$C_{18}H_{26}O_{10}$	447.149	$[M+HCOOH-H^+]^-$	425.14	$[M+Na^+]^+$	1.208	De Sorricellis et al., 2024
3	*A. lusitanicum*	Sinapic acid hexose	5.16	$C_{17}H_{22}O_{10}$	431.192	$[M+HCOOH-H^+]^-$	409.182	$[M+Na^+]^+$	-171.647	De Sorricellis et al., 2024
4	*A. lusitanicum*	Sinapic acid hexoside	5.35	$C_{17}H_{22}O_{10}$	385.112	$[M-H^+]^-$	409.107	$[M+Na^+]^+$	1.465	De Sorricellis et al., 2024
5	*A. lusitanicum*	UI	5.38	-	433.206	$[M-H^+]^-$	411.19	$[M+Na^+]^+$	-	De Sorricellis et al., 2024
6	*A. lusitanicum*	UI	5.58	-	433.207	$[M+HCOOH-H^+]^-$	411.199	$[M+Na^+]^+$	-	De Sorricellis et al., 2024
7	*A. lusitanicum*	Kaempferol-O-(O-hexosyl-hexosyl-desoxyhexoside)	5.74	$C_{33}H_{40}O_{20}$	755.203	$[M-H^+]^-$	757.218	$[M+H^+]^+$	-0.581	De Sorricellis et al., 2024
8	*A. lusitanicum*	Phenylethyl primeveroside	5.96	$C_{19}H_{28}O_{10}$	461.165	$[M+HCOOH-H^+]^-$	439.154	$[M+Na^+]^+$	-0.646	De Sorricellis et al., 2024
9	*A. lusitanicum*	Kaempferol-O-desoxyhexoside derivative	6.28	-	729.189	$[M-H^+]^-$	-	-	-	De Sorricellis et al., 2024
10	*A. lusitanicum*	UI	6.39	-	551.270	$[M+HCOOH-H^+]^-$	529.263	$[M+Na^+]^+$	-	De Sorricellis et al., 2024
11	*A. lusitanicum*	UI	6.65	-	553.289	$[M+HCOOH-H^+]^-$	531.279	$[M+Na^+]^+$	-	De Sorricellis et al., 2024
12	*A. lusitanicum*	UI	6.73	-	553.286	$[M+HCOOH-H^+]^-$	531.279	$[M+Na^+]^+$	-	De Sorricellis et al., 2024
13	*A. lusitanicum*	Saponin class IV4 + 2 hexoses + 1 desoxyhexose	7.06	$C_{45}H_{74}O_{19}$	963.483	$[M+HCOOH-H^+]^-$	941.47	$[M+Na^+]^+$	-3.114	De Sorricellis et al., 2024
14	*A. lusitanicum*	Saponin class IV4 + 2 hexoses + 1 desoxyhexose	7.13	$C_{45}H_{74}O_{19}$	963.481	$[M+HCOOH-H^+]^-$	941.47	$[M+Na^+]^+$	-1.451	De Sorricellis et al., 2024

15	*A. lusitanicum*	Saponin class V3 + 3 hexoses + 3 desoxyhexoses	7.42	$C_{62}H_{102}O_{32}$	678.325	$[2M-H^+]^-$	1381.656	$[M+Na^+]^+$	-23.588	De Sorricelis et al., 2024
16	*A. lusitanicum*	Saponin class IV4 + 2 hexoses + 1 desoxyhexose + acetyl group	7.60	$C_{47}H_{76}O_{20}$	1005.492	$[M+HCOOH-H^+]^-$	983.483	$[M+Na^+]^+$	-1.989	De Sorricellis et al., 2024
17	*A. lusitanicum*	Saponin class IV4 + 2 hexoses + 1 desoxyhexose	7.65	$C_{47}H_{76}O_{20}$	1005.492	$[M+HCOOH-H^+]^-$	983.483	$[M+Na^+]^+$	-1.989	De Sorricellis et al., 2024
18	*A. lusitanicum*	UI saponin	8.33	-	1241.580	$[M+HCOOH-H^+]^-$	1219.561	$[M+Na^+]^+$	-	De Sorricellis et al., 2024
19	*A. lusitanicum*	Saponin class IV3 + 2 hexoses + 2 desoxyhexoses + 1 pentose	8.44	$C_{56}H_{92}O_{26}$	1225.592	$[M+HCOOH-H^+]^-$	1203.575	$[M+Na^+]^+$	-7.343	De Sorricellis et al., 2024
20	*A. lusitanicum*	Saponin class IV3 + 2 hexoses + 2 desoxyhexoses	8.46	$C_{51}H_{84}O_{22}$	1093.545	$[M+HCOOH-H^+]^-$	1071.536	$[M+Na^+]^+$	-1.554	De Sorricellis et al., 2024
21	*A. lusitanicum*	Saponin class IV3 + 2 hexoses + 3 desoxyhexoses	8.52	$C_{57}H_{94}O_{26}$	1193.598	$[M-H^+]^-$	1217.597	$[M+Na^+]^+$	-2.513	De Sorricellis et al., 2024
22	*A. lusitanicum*	Saponin class IV3 + 2 hexoses + 1 desoxyhexose + 1 pentose	8.55	$C_{50}H_{82}O_{22}$	1079.531	$[M+HCOOH-H^+]^-$	1057.522	$[M+Na^+]^+$	-3.705	De Sorricellis et al., 2024
23	*A. lusitanicum*	Saponin class IV3 + 2 hexoses + 1 desoxyhexoses	8.65	$C_{45}H_{74}O_{18}$	947.487	$[M+HCOOH-H^+]^-$	925.479	$[M+Na^+]^+$	-1.785	De Sorricellis et al., 2024
24	*A. lusitanicum*	Saponin class III3 + 1 pentose + 1 hexose +3 desoxyhexoses	8.76	$C_{56}H_{90}O_{25}$	1207.573	$[M+HCOOH-H^+]^-$	1185.564	$[M+Na^+]^+$	0.816	De Sorricellis et al., 2024
25	*A. lusitanicum*	Saponin class IV3 + 2 hexoses + 3 desoxyhexoses + acetyl group	9.41	$C_{59}H_{96}O_{27}$	1281.611	$[M+HCOOH-H^+]^-$	1259.59	$[M+Na^+]^+$	-0.039	De Sorricellis et al., 2024
26	*S. pratensis*	Protocatechuic acid 4-O-β-hexoside	3.41	$C_{13}H_{16}O_9$	315.0716	$[M-H^+]^-$	339.1044	$[M+Na^+]^+$	0.00	152.0115; 108.0222
27	*S. pratensis*	UI	3.69		435.1145	$[M+HCOOH-H^+]^-$	413.1060	$[M+Na^+]^+$	-	389.1100; 227.0574; 209.0459; 165.0558; 141.0191
28	*S. pratensis*	Neochlorogenic acid	3.96	$C_{16}H_{18}O_9$	353.0877	$[M-H^+]^-$	377.0838	$[M+Na^+]^+$	-1.13	191.0566; 179.0365; 135.0465
29	*S. pratensis*	Esculetin	4.20	$C_{15}H_{16}O_9$	339.0719	$[M-H^+]^-$	341.0855	$[M+H^+]^+$	-0.88	177.0185; 133.0301
30	*S. pratensis*	Eucommicin A	4.22	$C_{32}H_{36}O_{18}$	707.1833	$[M-H^+]^-$	731.1789	$[M+Na^+]^+$	-1.41	191.06
31	*S. pratensis*	8-epiLoganic acid	4.28	$C_{16}H_{24}O_{10}$	375.1291	$[M-H^+]^-$	399.1259	$[M+Na^+]^+$	1.87	213.0777; 169.0873; 151.0768; 125.0611; 113.0245

32	*S. pratensis*	Chlorogenic acid	4.63	$C_{16}H_{18}O_9$	353.0887	$[M-H^+]^-$	377.0834	$[M+Na^+]^+$	-3.97	191.06
33	*S. pratensis*	Oleoside	4.74	$C_{16}H_{22}O_{11}$	389.1089	$[M-H^+]^-$	-	-	-1.28	345.1196; 209.0459; 183.0653; 165.0558; 121.0653
34	*S. pratensis*	Caffeoyl quinic acid isomer	4.80	$C_{16}H_{18}O_9$	353.0875	$[M-H^+]^-$	377.0836	$[M+Na^+]^+$	-0.57	191.0566; 179.0338; 173.0463; 135.0441
35	*S. pratensis*	Swertiamarin	4.90	$C_{16}H_{22}O_{10}$	419.1187	$[M+HCOOH-H^+]^-$	397.1110	$[M+Na^+]^+$	0.72	373.1129; 355.1029; 193.0505; 179.0557; 161.0469; 149.0610; 141.0191; 119.0359; 113.0245; 101.0241; 89.0237
36	*S. pratensis*	Luteolin-C-hexosyl-O-hexoside	5.12	$C_{27}H_{30}O_{16}$	609.1457	$[M-H^+]^-$	611.1613	$[M+H^+]^+$	-0.16	447.0943; 357.0610; 313.0364; 299.0557; 285.0418
37	*S. pratensis*	Gentiopicroside	5.32	$C_{16}H_{20}O_9$	401.1083	$[M+HCOOH-H^+]^-$	379.1000	$[M+Na^+]^+$	0.25	355.1029; 193.0505; 179.0557; 161.0469; 149.0610; 119.0359; 101.0241; 89.0237
38	*S. pratensis*	Sweroside	5.41	$C_{16}H_{22}O_9$	403.1238	$[M+HCOOH-H^+]^-$	381.1157	$[M+Na^+]^+$	0.50	357.1190; 195.0662; 179.0557; 161.0469; 151.0768; 125.0245; 119.0359; 113.0245
39	*S. pratensis*	Apigenin-C-hexosyl-O-hexoside	5.58	$C_{27}H_{30}O_{15}$	593.1515	$[M-H^+]^-$	595.1666	$[M+H^+]^+$	-1.52	431.0982; 311.0552; 297.0417; 283.0619; 269.0463
40	*S. pratensis*	Luteolin-C-hexosy-O-(caffeoyl)hexoside	5.74	$C_{36}H_{36}O_{19}$	771.1782	$[M-H^+]^-$	773.1932	$[M+H^+]^+$	-1.17	447.0943; 357.0610; 327.0514; 299.0557; 285.0418; 179.0392; 161.0235
41	*S. pratensis*	Apigenin-C-hexosyl-O-(caffeoyl)hexoside	6.13	$C_{36}H_{36}O_{18}$	755.1823	$[M-H^+]^-$	757.1973	$[M+H^+]^+$	1.06	431.0982; 341.0671; 311.0552; 283.0619; 179.0392; 161.0235
42	*S. pratensis*	Dicaffeoyl quinic acid	6.96	$C_{25}H_{24}O_{12}$	515.1199	$[M-H^+]^-$	-	-	-1.75	353.0887; 191.0566; 179.0463
43	*S. pratensis*	Dicaffeoyl quinic acid	7.24	$C_{25}H_{24}O_{12}$	515.1188	$[M-H^+]^-$	-	-	0.39	353.0887; 191.0566; 179.0365; 173.0463; 135.0465
44	*S. pratensis*	Akebia saponin D or Palustroside III	8.81	$C_{47}H_{76}O_{18}$	973.4997	$[M+HCOOH-H^+]^-$	951.4901	$[M+Na^+]^+$	1.13	927.5005; 603.3918; 573.3791; 323.0957; 179.0557
45	*S. pratensis*	Akebia saponin D or Palustroside III isomer	9.53	$C_{47}H_{76}O_{18}$	927.4968	$[M-H^+]^-$	951.4901	$[M+Na^+]^+$	-1.62	603.3918; 323.0957; 179.0611
46	*S. pratensis*	4′-O-Acetyl-akebia saponin D or acetyl-palustroside III	9.97	$C_{49}H_{78}O_{19}$	1015.5114	$[M+HCOOH-H^+]^-$	993.5025	$[M+Na^+]^+$	2.07	969.5089; 645.4037; 627.3903; 323.0994
47	*D. superbus*	UI	2.58		349.0769	$[M+HCOOH-H^+]^-$	327.0689	$[M+Na^+]^+$	-	303.072; 141.0206; 111.0095

48	*D. superbus*	UI	2.90		309.1195	[M+HCOOH-H$^+$]$^-$	287.1102	[M+Na$^+$]$^+$	-	174.9536; 146.9610
49	*D. superbus*	UI	3.57		291.1080	[M-H$^+$]$^-$	315.1053	[M+Na$^+$]$^+$	-	161.0457; 113.0240; 101.0217
50	*D. superbus*	UI	3.49		499.1656	[M+HCOOH-H$^+$]$^-$	477.1580	[M+Na$^+$]$^+$	-	453.1626; 263.0778; 221.0660; 179.0569; 161.0457; 143.0352
51	*D. superbus*	UI	3.60		337.1133	[M+HCOOH-H$^+$]$^-$	315.1049	[M+Na$^+$]$^+$	-	291.1070; 161.8907; 243.8793; 225.8662; 207.8649; 189.0770; 161.0431; 101.0237
52	*D. superbus*	UI	3.95		453.1605	[M-H$^+$]$^-$	477.1578	[M+Na$^+$]$^+$	-	291.1070; 189.0770; 179.0569; 161.0431; 143.0328; 119.0353; 113.0240; 101. 0237; 89.0255
53	*D. superbus*	UI	4.44		447.1863	[M+HCOOH-H$^+$]$^-$	425.1777	[M+Na$^+$]$^+$	-	401.1791; 249.0975; 239.1277; 221.1178; 153.0923
54	*D. superbus*	Tetrahydroxy-flavone-C-hexoside-O-hexoside (luteolin)	5.13	$C_{27}H_{30}O_{16}$	609.1469	[M-H$^+$]$^-$	611.1613	[M+H$^+$]$^+$	-2.13	447.0967; 357.0603; 327.0511; 313.0363; 299.0557; 285.0420
55	*D. superbus*	Tetrahydroxy-flavone-C-(deoxyhexosyl)hexoside-O-hexoside (luteolin)	5.13	$C_{33}H_{40}O_{20}$	801.2114	[M+HCOOH-H$^+$]$^-$	757.2191	[M+H$^+$]$^+$	-3.12	593.1556; 473.1092; 429.0804; 369.0630; 357.0641; 339.0550; 327.0511; 309.0406; 298.0479; 285.0420
56	*D. superbus*	Trihydroxy-flavone-C-(deoxyhexosyl)hexoside-O-hexoside (apigenin)	5.57	$C_{33}H_{40}O_{19}$	739.2106	[M-H$^+$]$^-$	741.2238	[M+H$^+$]$^+$	-2.71	577.1600; 413.0885; 293.0447
57	*D. superbus*	Trihydroxy-flavone derivative (apigenin)	5.78	$C_{28}H_{32}O_{16}$	623.1624	[M-H$^+$]$^-$	647.1552	[M+Na$^+$]$^+$	-1.93	461.1047; 445.0737; 341.0628; 327.0511; 298.0479; 269.0466
58	*D. superbus*	Tetrahydroxy-flavone derivative (luteolin)	5.86	$C_{41}H_{46}O_{23}$	905.2353	[M-H$^+$]$^-$	907.2501	[M+H$^+$]$^+$	-0.11	593.1508; 473.1069; 429.0814; 357.0590; 339.0481; 327.0497; 309.0396; 285.0380
59	*D. superbus*	Tetrahydroxy-flavone derivative (luteolin)	6.25	$C_{43}H_{48}O_{23}$	931.2508	[M-H$^+$]$^-$	933.2654	[M+H$^+$]$^+$	0.00	593.1508; 473.1069; 429.0814; 357.0590; 339.0481; 327.0497; 309.0396; 285.0380
60	*D. superbus*	UI	6.40		565.1918	[M+HCOOH-H$^+$]$^-$	543.1840	[M+Na$^+$]$^+$	-	339.1266; 327.1214
61	*D. superbus*	UI	6.59		1195.5383	[M+HCOOH-H$^+$]$^-$	1173.5278	[M+Na$^+$]$^+$	-	1149.537; 969.473; 663.277; 485.146; 439.321
62	*D. superbus*	Akebonoic acid + 2 hexose derivative	6.61	$C_{47}H_{74}O_{19}$	987.4818	[M-H$^+$]$^-$	1011.4756	[M+Na$^+$]$^+$	-1.76	663.3770; 439.3196

63	*D. superbus*	Tetrahydroxy-flavone derivative (luteolin)	6.94	$C_{41}H_{46}O_{23}$	951.2431	[M+HCOOH-H+]-	907.2502	[M+H+]+	-2.62	905.2375; 743.1837; 579.1136; 473.1048; 441.0736; 357.0641; 327.0511; 309.0406; 298.0514; 285.0420
64	*D. superbus*	Tetrahydroxy-flavone derivative (luteolin)	7.55	$C_{44}H_{52}O_{25}$	979.2747	[M+HCOOH-H+]-	935.2810	[M+H+]+	-2.83	933.2717; 771.2173; 607.1489; 575.1409; 473.1092; 411.0694; 357.0603; 327.0511; 309.0406; 298.0443; 285.0385
65	*D. superbus*	Tetrahydroxy-flavone-C-deoxyhexose-O-hexoside derivative (luteolin)	7.58	$C_{43}H_{50}O_{23}$	979.2736	[M+HCOOH-H+]-	935.2813	[M+H+]+	-1.74	933.2717; 771.2173; 607.1489; 575.1409; 473.1092; 411.0694; 369.0591; 357.0603; 339.0512; 327.0511; 309.0406; 298.0443; 285.0385
66	*D. superbus*	Tetrahydroxyflavone-C-(feruloyl+deoxyhexosyl)hexoside-O-hexoside	7.65	$C_{43}H_{48}O_{23}$	931.2538	[M-H+]-	933.2620	[M+H+]+	0.59	298.0443; 285.0316; 193.0516; 309.0406; 473.1092; 575.1409; 593.151; 605.133; 769.201; 931.253
67	*D. superbus*	Trihydroxy-flavone derivative (apigenin)	7.95	$C_{43}H_{50}O_{22}$	963.2808	[M+HCOOH-H+]-	919.2868	[M+H+]+	-3.94	917.2724; 755.2221; 591.1535; 559.1449; 457.1141; 395.072; 293.0447; 269.0433; 195.0645
68	*D. superbus*	Gypsogenic acid + 5 hexose	8.22	$C_{60}H_{96}O_{30}$	1295.5931	[M-H+]-	1319.5860	[M+Na+]+	-1.81	1115.5347; 647.3813; 485.1463; 467.1407; 423.3257; 383.1207; 221.0691; 179.0569; 161.0431
69	*D. superbus*	Tetrahydroxy-flavone derivative (luteolin)	8.49	$C_{53}H_{60}O_{26}$	1111.3313	[M-H+]-	1113.3430	[M+H+]+	-1.63	771.2173; 607.1489; 575.1458; 473.1092; 411.0694; 357.0680; 327.0511; 309.0406; 298.0479; 285.0479
70	*D. superbus*	Gypsogenic acid + 5 hexose	8.64	$C_{60}H_{96}O_{30}$	1295.5927	[M-H+]-	1319.5790	[M+Na+]+	-1.45	1115.5415; 647.3813; 485.1463; 467.1452; 423.3257; 383.1167; 221.0630; 179.0542
71	*D. superbus*	Gypsogenic acid + 4 hexoses	8.80	$C_{54}H_{86}O_{25}$	1179.5459	[M+HCOOH-H+]-	1157.5350	[M+Na+]+	-2.00	1133.5435; 971.4943; 953.4749; 647.3813; 485.1508; 423.3300
72	*D. superbus*	Gypsogenic acid + 3 hexoses	8.95	$C_{48}H_{76}O_{20}$	971.4882	[M-H+]-	995.4840	[M+Na+]+	-3.11	647.3813; 423.3257
73	*D. superbus*	Gypsogenic acid + 3 hexoses + 1 pentose	9.26	$C_{53}H_{84}O_{24}$	1149.5324	[M+HCOOH-H+]-	1127.5220	[M+Na+]+	3.49	1103.5295; 923.4627; 701.3907; 617.3733; 503.3363; 423.3300; 305.0849
74	*D. superbus*	Polygalacic acid + 3 hexoses	9.52	$C_{48}H_{78}O_{21}$	989.4984	[M-H+]-	1013.4920	[M+Na+]+	-2.73	503.336; 485.3312; 457.3329; 441.3381

75	*D. superbus*	Gypsogenic acid hexose derivative	9.63	$C_{48}H_{77}O_{20}$	1039.4749	$[M+HCOOH-H^+]^-$	995.4770	$[M+H^+]^+$	0.10	993.4719; 971.4943; 729.3781; 507.3112; 485.1508; 323.0954; 179.0542
76	*D. superbus*	Gypsogenic acid + 3 hexoses	9.64	$C_{48}H_{76}O_{20}$	971.4881	$[M-H^+]^-$	995.4811	$[M+H^+]^+$	-2.99	485.33
77	*D. superbus*	Polygalacic acid + 2 hexoses	9.79	$C_{42}H_{68}O_{16}$	827.4449	$[M-H^+]^-$	851.4390	$[M+Na^+]^+$	-2.42	503.3363; 485.3267; 457.3373; 441.3381
78	*D. superbus*	Gypsogenic acid + 2 hexoses	9.96	$C_{42}H_{66}O_{15}$	809.4349	$[M-H^+]^-$	833.4270	$[M+Na^+]^+$	-3.21	485.33
79	*D. superbus*	Gypsogenic acid derivative	10.31	$C_{48}H_{76}O_{20}$	1017.4923	$[M+HCOOH-H^+]^-$	995.4770	$[M+Na^+]^+$	-1.69	971.4879; 791.4225; 569.3491; 485.3267; 305.0884; 179.0569
80	*D. superbus*	UI	11.43		1699.7247	$[M-H^+]^-$	1723.7180	$[M+Na^+]^+$	-	955.46

Figure S1

A

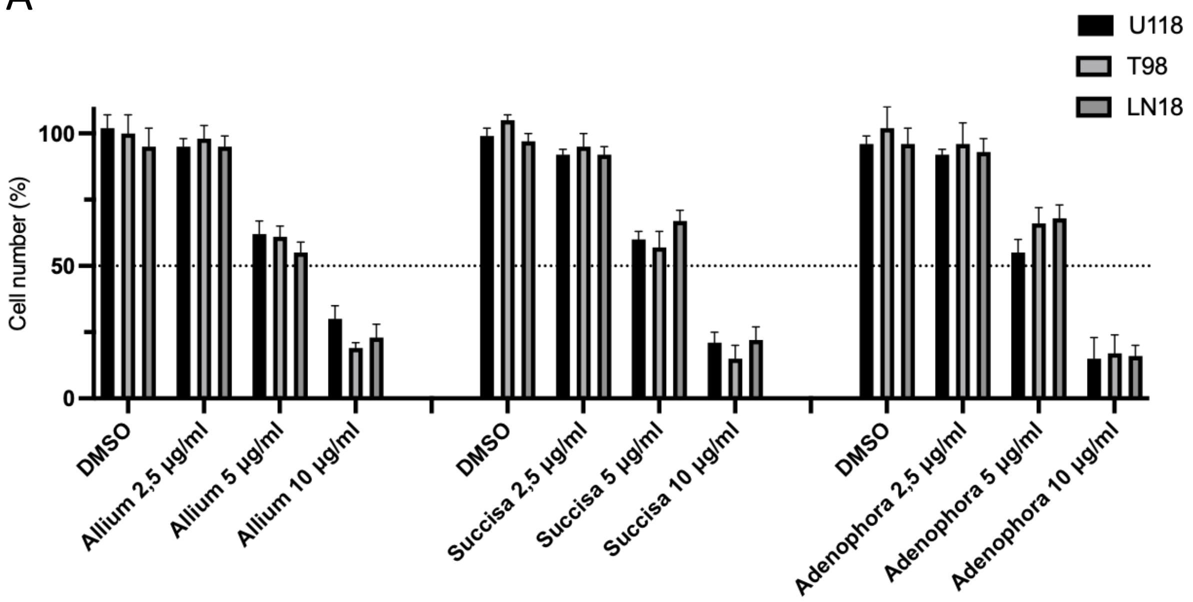
U118
T98
LN18
Cell number (%)
100
50
0
DMSO
Allium 2,5 µg/ml
Allium 5 µg/ml
Allium 10 µg/ml
DMSO
Succisa 2,5 µg/ml
Succisa 5 µg/ml
Succisa 10 µg/ml
DMSO
Adenophora 2,5 µg/ml
Adenophora 5 µg/ml
Adenophora 10 µg/ml

B

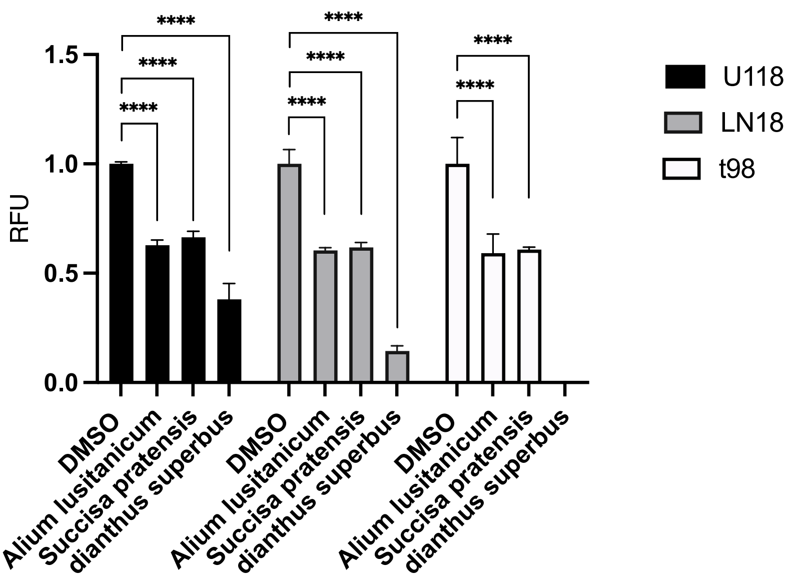
Cell proliferation
U118
LN18
t98
RFU
1.5
1.0
0.5
0.0
****
DMSO
Alium lusitanicum
Succisa pratensis
dianthus superbus

C

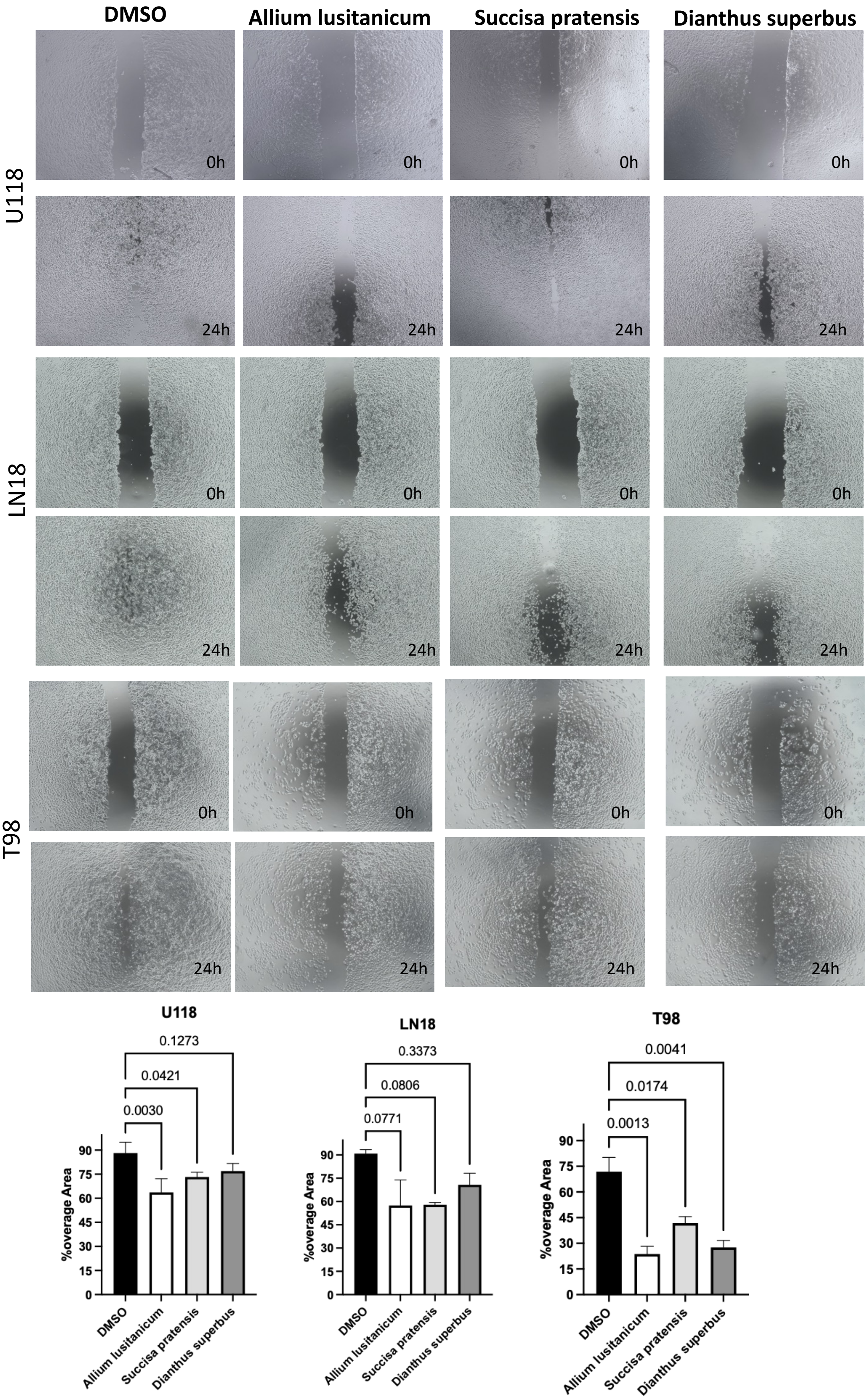
DMSO
Allium lusitanicum
Succisa pratensis
Dianthus superbus
U118
LN18
T98
0h
24h
U118
0.1273
0.0421
0.0030
LN18
0.3373
0.0806
0.0771
T98
0.0041
0.0174
0.0013
%overage Area
DMSO
Allium lusitanicum
Succisa pratensis
Dianthus superbus

D

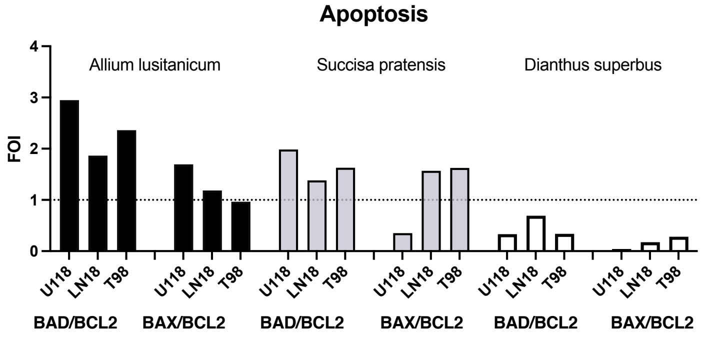
Apoptosis
Allium lusitanicum
Succisa pratensis
Dianthus superbus
FOI
U118
LN18
T98
BAD/BCL2
BAX/BCL2
BAD/BCL2
BAX/BCL2
BAD/BCL2
BAX/BCL2

E

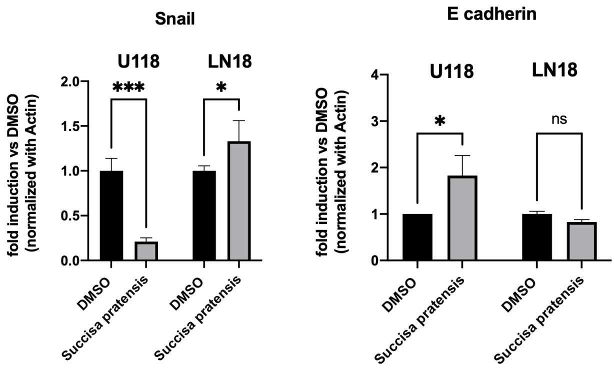
Snail
U118
LN18
***
*
E cadherin
U118
LN18
*
ns
fold induction vs DMSO
(normalized with Actin)
DMSO
Succisa pratensis

F

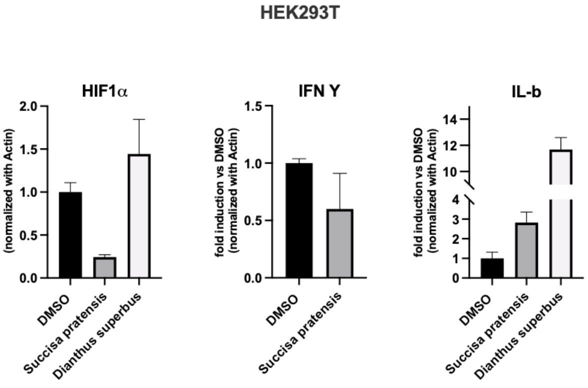
HEK293T
HIF1α
IFN Y
IL-b
fold induction vs DMSO
(normalized with Actin)
DMSO
Succisa pratensis
Dianthus superbus

Figure S2

A

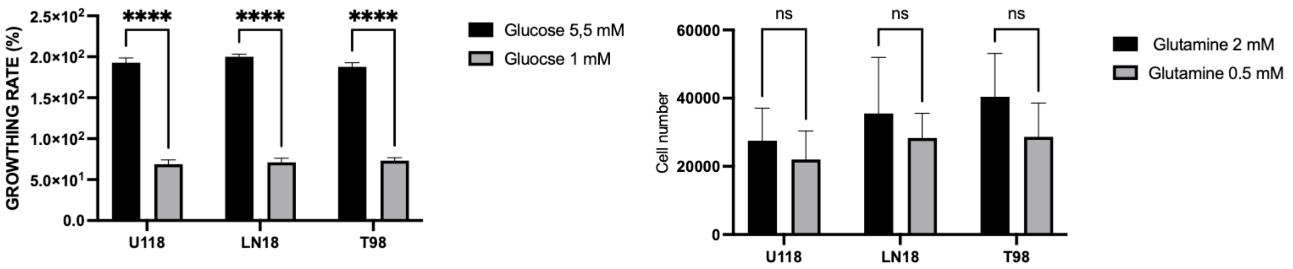
****
****
****
2.5×10²
2.0×10²
1.5×10²
1.0×10²
5.0×10¹
0.0
GROWTHING RATE (%)
U118
LN18
T98
Glucose 5,5 mM
Gluocse 1 mM
ns
ns
ns
60000
40000
20000
0
Cell number
U118
LN18
T98
Glutamine 2 mM
Glutamine 0.5 mM

B

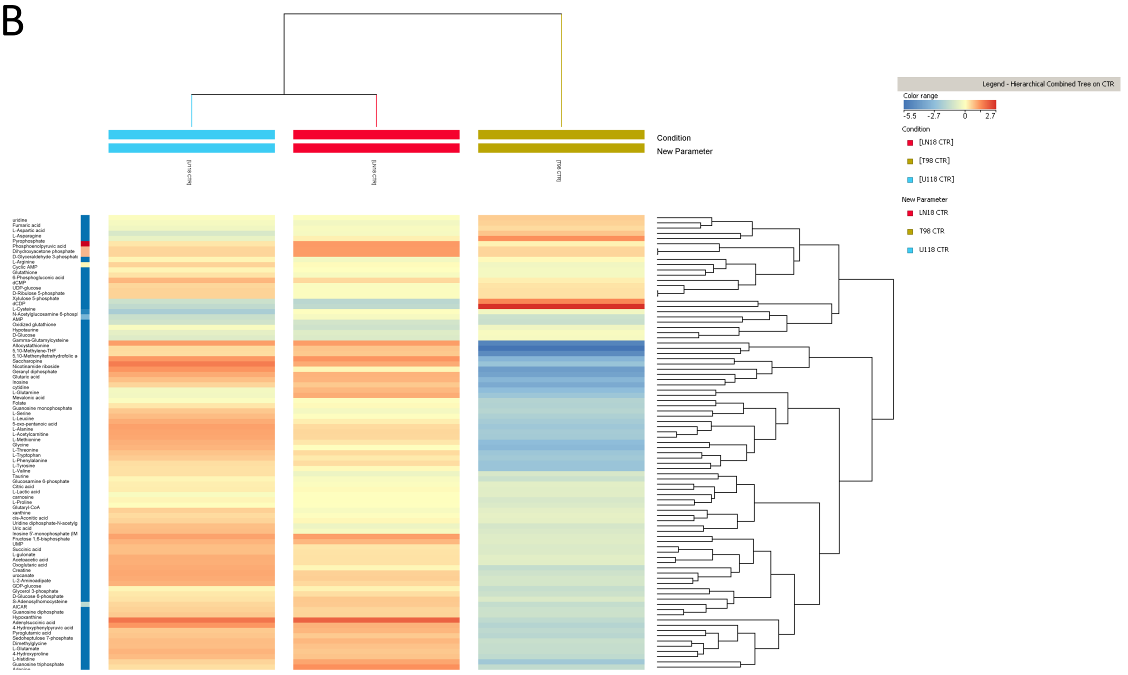
Condition
New Parameter
Legend - Hierarchical Combined Tree on CTR
Color range
-5.5
-2.7
0
2.7
Condition
[LN18 CTR]
[T98 CTR]
[U118 CTR]
New Parameter
LN18 CTR
T98 CTR
U118 CTR

SUPPLEMENTARY TABLES

Supplementary Table S2. List of primers used for qPCR.

Gene	Primer sequence Fw	Primer sequence Rw
β-actin	CACCATTGGCAATGAGCGGTTC	AGGTCTTTGCGGATGTCCACG
HIF-1α	TGATTGCATCTCCATCTCCTACC	GACTCAAAGCGACAGATAACACG
IFNγ	GAGTGTGGAGACCATCAAGGAAG	TGCTTTGCGTTGGACATTCAAGTC
IL-18	GATAGCCAGCCTAGAGGTATGG	CCTTGATGTTATCAGGAGGATTCA
IL-1b	CCACAGACCTTCCAGGAGAATG	GTGCAGTTCAGTGATCGTACAGG
NFkB	GCAGCACTACTTCTTGACCACC	TCTGCTCCTGAGCATTGACGTC
E-CAD	GCTGAGCTGGACAGGGAGGA	ATGGGGGCGTTGTCATTCAC
NRF2	CAGCGACGGAAAGAGTATGA	TGGGCAACCTGGGAGTAG
KEAP1	TTCTGGGGATCCATG	CTCCAAGGACGTAGATTCTC
GPX4	CGCAACGATGTTGCCTGGAACTTT	AGGCTCGATGTCAATGGTCTGGAA
BAD	CCAACCTCTGGGCAGCACAGC	TTTGCCGCATCTGCGTTGCTGT
BAX	TCAGGATGCGTCCACCAAGAAG	TGTGTCCACGGCGGCAATCATC
BCL-2	ATCGCCCTGTGGATGACTGAGT	GCCAGGAGAAATCAAACAGAGGC
SOD-1	CTCACTCTCAGGAGACCATTGC	CCACAAGCCAAACGACTTCCAG
SOD-2	CTGGACAAACCTCAGCCCTAAC	AACCTGAGCCTTGGACACCAAC
Catalase	GTGCGGAGATTCAACACTGCCA	CGGCAATGTTCTCACACAGACG
SNAIL	GAAAGGCCTTCAACTGCAAA	TGACATCTGAGTGGGTCTGG
